# Economic Burden of West Nile Virus Disease, Quebec, Canada, 2012–2013

**DOI:** 10.3201/eid2510.181608

**Published:** 2019-10

**Authors:** Najwa Ouhoummane, Eric Tchouaket, Anne-Marie Lowe, Ann Fortin, Dahlia Kairy, Anne Vibien, Jessica Kovitz-Lensch, Terry-Nan Tannenbaum, François Milord

**Affiliations:** Institut National de Santé Publique du Quebec, Quebec City, Canada (N. Ouhoummane, A. Fortin, F. Milord);; Université du Quebec en Outaouais, Gatineau, Quebec, Canada (E. Tchouaket);; Public Health Agency of Canada, Montreal, Quebec, Canada (A.-M. Lowe);; Université de Montreal, Montreal, Quebec, Canada (D. Kairy);; Centre de Recherche Interdisciplinaire en Réadaptation du Montreal Métropolitain, Montreal (D. Kairy);; Centre Intégré de Santé et de Services Sociaux de la Montérégie Est-Hôpital Honoré Mercier, St.-Hyacinthe, Quebec, Canada (A. Vibien, J. Kovitz-Lensch);; Institut National de Santé Publique de Montreal, Montreal (T.-N. Tannenbaum);; Université de Sherbrooke, Sherbrooke, Quebec, Canada (F. Milord)

**Keywords:** West Nile virus disease, West Nile encephalitis, West Nile fever, West Nile meningitis, West Nile virus, WNV, viruses, economic burden, costs, meningitis/encephalitis, zoonoses, Quebec, Canada

## Abstract

The economic burden of West Nile virus (WNV) infection is not known for Canada. We sought to describe the direct and indirect costs of WNV infection in the province of Quebec, Canada, up to 2 years after onset of signs and symptoms. We conducted a retrospective cohort study that included WNV cases reported during 2012 and 2013. For 90 persons infected with WNV, persons with encephalitis accounted for the largest proportion of total cost: a median cost of $21,332 per patient compared with $8,124 for West Nile meningitis (p = 0.0004) and $192 for West Nile fever (p<0.0001). When results were extrapolated to all reported WNV patients, the estimated total cost for 124 symptomatic cases was ≈$1.7 million for 2012 and that for 31 symptomatic cases was ≈$430,000 for 2013. Our study provides information for the government to make informed decisions regarding public health policies and infectious diseases prevention and control programs.

West Nile virus (WNV) infection is endemic to North America. More than 41,000 cases of WNV-related illnesses and 2,000 deaths were reported in the United States between the introduction of the virus in 1999 and 2015 (*1*). During the same period, 5,310 cases were reported in Canada (*2*). In Quebec, the first cases were documented in 2002. After a quiet period (2004–2010), this province experienced an outbreak in 2012 (124 symptomatic cases); since 2013, the number of cases has remained stable (average of 30 cases/year) (*3*).

WNV causes an asymptomatic infection in 80% of cases, but in <1% of cases, a severe illness occurs with neurologic involvement, such as aseptic meningitis, encephalitis, or acute flaccid paralysis (*4*,*5*). WNV infection and particularly neurologic disease have been associated with mild to severe clinical manifestations, might require hospitalization, and lead to long-term sequelae and death (*6*).

To date, 3 studies in the United States have estimated the economic burden of WNV disease (*7*–*9*). These estimates can be used in assessing the cost-effectiveness of various interventions designed to decrease WNV disease risk (*8*). In the province of Quebec, Canada, a cost-effectiveness analysis was conducted during 2006 (*10*). However, this study was based on a hypothetical simulation of 2 scenarios (high activity of the virus versus low-activity season); therefore, the cost estimations could be speculative. Thus, in Canada, no data are available on the actual costs of WNV disease, and results from the United States cannot be extrapolated because of differences in the organization of the heathcare systems and costs, WNV disease prevention programs, mean income, and standards of living. Furthermore, exchange rate differences do not accurately reflect real differences in purchasing power (*11*). The objective of this study was to estimate direct and indirect costs of WNV disease cases in the province of Quebec, Canada, up to 2 years after symptom onset.

## Materials and Methods

### Study Population

WNV infection is a reportable disease in Quebec. Physicians and laboratories must report all WNV-positive cases to regional public health boards, conduct which epidemiologic investigations to document the infection; determine the likely place of acquisition; and collect sociodemographic and clinical information, such as date of illness onset and clinical syndrome (i.e., uncomplicated fever, meningitis, encephalitis, and acute flaccid paralysis). The data are entered into the integrated system for public health monitoring of West Nile virus, a provincial electronic surveillance system for WNV disease that includes information on humans, mosquitoes, and animals (*12*). During 2012–2013, a total of 155 symptomatic WNV cases were reported in Quebec.

We asked regional public health boards to contact each of the case-patients to see whether they were willing to participate in a study. A research nurse obtained written consent from all eligible case-patients to participate in a telephone interview and to enable access to their medical charts. Consent was obtained from parents or family of patients who were <18 years of age and for patients who had died. More details on the methods are available in a previous article (*6*). The study protocol was submitted to the public health research ethics board of the Institut National de Santé Publique du Quebec, which provided a favorable recommendation for the study (*13*).

### Case Definitions

A WNV-positive case includes a laboratory diagnosis of WNV infection by IgM capture enzyme immunoassays for either serum or cerebrospinal fluid samples. In Quebec, after a first IgM-positive case has been confirmed by using a plaque reduction neutralization test, all other IgM-positive cases in the same season are considered to be laboratory confirmed. Cases are further classified according to their clinical manifestations: West Nile fever (WNF, an acute systemic febrile illness), West Nile meningitis (WNM, with stiff neck and cerebrospinal fluid pleocytosis), West Nile encephalitis (WNE, with altered mental status), West Nile meningoencephalitis, and acute flaccid paralysis (AFP, polio-like myelitis, or Guillain-Barré syndrome) (*14*).

### Data Collection

For all eligible patients, we administered a telephone questionnaire 24 months after sign or symptom onset to document medical service, productivity losses, and expenses up to 2 years after the acute phase of WNV disease. We developed the questionnaire on the basis of the study by Staples et al. (*8*). Participants were asked to document the following items related to their WNV disease after initial hospitalization or consultation: subsequent hospitalization, stay in a rehabilitation center, physiotherapy, occupational therapy, speech therapy, home care, primary care physician consultations, neurologist consultations, medications, medical equipment, recruitment for household chores, and all other personal expenses incurred by the WNV disease. We also considered time off from work for the patient and for family members (to care for the patient). For hospitalized patients, we obtained data for hospital stay, intensive care unit admission, and inpatient rehabilitation from medical records.

#### Costs Estimation

We based the estimation of costs on the principle of human capital and a societal perspective (relevant costs regardless of who paid) (*15*). For their initial care, eligible patients either were hospitalized (n = 71), were seen in an emergency department but not hospitalized (n = 10), or consulted with doctor in a clinic (n = 12). For the third group, we did not collect any costs associated with their initial care.

#### Hospitalization

We obtained costs for initial hospitalization (no subsequent hospitalization was reported) by using the All Patient Refined–Diagnosis-Related Groups (APR-DRG) and the relative use of resources. The APR-DRG provides average costs incurred for inpatient services. These costs include all inpatient allied healthcare, surgical and medical procedures, medication, and laboratory tests. Because permission to access individual costs data was not obtained, we created 19 groups of patients on the basis of 4 criteria: the principal diagnosis of each patient (by using the International Classification of Diseases, 10th Revision), patient’s age (<60 vs. >60 years of age), admission to intensive care unit (yes or no), and diagnostic year (2012 or 2013). Nearly 60% of hospitalized participants had specific principal International Classification of Diseases, 10th Revision, diagnosis code of WNV (A923) (Table 1). For other participants with no specific principal diagnosis code of WNV, WNV infection had to be coded as secondary diagnosis in the APR-DRG database to ensure specificity. The mean and median costs in the APR-DRG for each group were attributed to each patient in the group.

**Table 1:** Principal diagnoses for 71 hospitalized case-patients with West Nile virus infection, Quebec, Canada*

Principal diagnosis	ICD-10 code	No. (%) patients
West Nile virus infection	A923	42 (59)
Viral meningitis, unspecified	A879	16 (23)
Viral infection, unspecified	B349	7 (10)
Unspecified viral encephalitis	A86	3 (4)
Guillain–Barré syndrome	G610	2 (3)
Headache	R51	1 (1)

*ICD-10, International Classification of Diseases, 10th Revision.

#### Inpatient Rehabilitation

Cost for inpatient rehabilitation was based on the average daily cost of services in a rehabilitation center for physical disabilities in 2012–2013. This factor was multiplied by the number of days spent in a center, which was obtained from the medical record.

#### Emergency Department Consultations

For patients seen in an emergency department, the physician service claims database was used to estimate the cost of medical consultations in the emergency department (Canadian $109.90) (*16*). This rate includes only the physician remuneration. Costs associated with emergency department stays and laboratory examinations and accommodations were not included.

#### Medical and Paramedical Care up to 2 Years after Sign/Symptom Onset

During the telephone interview, patients were asked to provide information on types and numbers and duration of outpatient medical and paramedical services that they sought after the acute-care period. We obtained estimated costs for these services by multiplying type-specific cost estimates by the number of visits reported (Table 2) (*16*). The number of follow-up physician visits was missing for 2 participants; a minimum of 1 visit was used for those case-patients.

**Table 2:** Physician service claims billing codes and costs for outpatient paramedical services, Quebec, Canada, 2012–2013*

Medical/paramedical service	Régie de l’Assurance maladie du Québec billing code	Patient age, y: rate in 2014/visit
Primary care physician visit	08874	<60: $40.00
	00074	60–69: $42.05
	08879	70–79: $48.60
	08881	>80: $50.80
Neurologist visit	09162: initial visit	$70.60
	09164: control visit	$40.00
Physiotherapy, occupational and speech therapy	None	$79.00
Home care	09171	$44.00

*Costs are in Canadian dollars.

#### Medical Equipment

Patients were asked about the acquisition of specific equipment and associated costs. Costs were assigned a value of 0 if the equipment was provided by government or was borrowed.

#### Recruitment for Household Chores

We obtained information for patients who needed aids for household chores. We also asked them to provide associated costs.

#### Absence from Work

We obtained information about missed workdays by patients or family members to care for a patient. Patients or family members having a job at their time of their WNV infection were asked to provide the occupation, the number of days they worked per week, and the number of days they missed work (including hospital stay). Income data for each patient or family member were obtained from the wage guide by occupation in Quebec according to their occupation. Productivity losses were estimated by multiplying the time taken off work by a weekly wage.

Occupation data were missing for 2 patients and 2 family members. For these 4 persons, we used the minimum wage. One patient reported stopping work because of his WNV infection. For this patient, we estimated the associated cost as the number of potential years of lost employment (65 minus age at infection). All persons (n = 11) who died during their initial hospitalization were >65 years of age. Therefore, productivity losses caused by death were not taken into account.

#### Other Personal Expenses

Patients were asked about other costs that they had to assume. For ambulance transportation, the basic cost of Canadian $125 was used.

### Data Analysis

For data analysis, we combined West Nile meningoencephalitis cases (n = 18) and AFP cases (n = 2) with WNE cases (n = 28) because of similar clinical manifestations; 1 case with missing information about clinical syndrome was excluded. All analyses were performed according to 3 clinical categories (WNF, WNM, and WNE). We compared proportions by using the χ^2^ test or Fisher exact test when appropriate. We compared distributions of age and hospital stay by using nonparametric tests (Wilcoxon rank-sum or Kruskal-Wallis tests).

Because cost distributions were not normal, we calculated mean and median values with the interquartile range (IQR). In this study, participants and nonparticipants were comparable with regard to demographic and illness severity (see Results). Thus, we assumed that participants were representative of the total number of WNV cases during 2012 and 2013, and we extrapolated estimated costs to all reported WNV cases according to clinical syndrome, cost category, and year. For each category, we estimated total number of cases (except for hospitalization, for which the exact number of cases was known) and total cost. For example, the total number of WNE case-patients admitted to inpatient rehabilitation (N_t_) = total number of WNE case-patients × proportion of WNE case-patients admitted to inpatient rehabilitation. The total cost for this category = N_t_ × C_p_, in which C_p_ is the inpatient rehabilitation median costs per WNE case-patient.

We performed analyses by using SAS version 9.1 (SAS Institute Inc., https://www.sas.com). A 2-sided p<0.05 was considered statistically significant.

## Results

Of the 155 symptomatic WNV patients during 2012–2013, a total of 93 (60%) agreed to participate in the study, but 2 of them could not be reached for the telephone interview and 1 with missing information about clinical syndrome was excluded from analyses. Medical records were available for 81 (87%) patients (71 hospitalizatons and 10 emergency department visits). Participants and nonparticipants were comparable with regard to demographics (except for sex; more women agreed to participate [54% vs. 36%; p = 0.026]) and illness severity (hospitalization, clinical syndrome, and death).

### Demographic and Clinical Characteristics

We obtained demographic and clinical characteristics of patients (Table 3). For 90 patients, WNF accounted for 27%, WNM 20%, and WNE 53%. Patients with WNE were significantly older than patients with WNM or WNF; 71% of WNE patients were >60 years of age compared with 22% of WNM patients and 29% of WNF patients (p<0.0001). Most patients with neurologic syndrome were hospitalized. The median hospital stay was longer for WNE patients than for WNM patients (p<0.0001) or WNF patients (p = 0.010). Ten (21%) WNE patients and 1 (6%) WNM patient died during hospitalization. In addition, 3 WNE patients, 2 who were discharged to home with support and 1 requiring rehabilitation services, died during the follow-up period, but their deaths were not considered to be directly related to WNV infection.

**Table 3:** Demographic and clinical characteristics of 90 WNV patients by clinical syndrome, Quebec, Canada, 2012–2013*

Characteristic	WNF, n = 24	WNM, n = 18	WNE, n = 48†
Age group, y			
<50	7 (29)	7 (39)	6 (12)
50–59	10 (42)	7 (39)	8 (17)
≥60	7 (29)	4 (22)	34 (71)
Sex			
M	7 (29)	8 (44)	24 (50)
F	17 (71)	10 (56))	24 (50)
Hospitalization	5 (21)	17 (94)	47 (98)
Days hospitalized, median (range)	4 (2–12)	4 (1–36)	12 (3–663)
Intensive care	0	0	22/47 (47)
Complications	0	3/17(18)	21/47 (45)
Death in hospital	0	1/17 (6)	10/47 (21)

*Values are no. (%) or no. occurrences/no. hospitalized (%) unless otherwise indicated. WNE, West Nile encephalitis; WNF, West Nile fever; WNM, West Nile meningitis; WNV, West Nile virus. †Includes WNE (n = 28), meningoencephalitis (n = 18), and acute flaccid paralysis (n = 2).

### Use of Medical and Paramedical Services and Absence of Work

We obtained data for use of medical and paramedical services up to 2 years after the acute phase of WNV disease (Table 4). When we excluded in-hospital deaths, the most common services used by participants were physician visits (66%), followed by medications (41%) and neurologist visits (26%). In general, WNE and WNM patients used more services and WNM participants needed more physician (p = 0.016) and neurologist (p = 0.029) visits. Six WNE patients were admitted to rehabilitation (median length of stay 75 days, range 30–120 days). Most patients who had a job missed work because of their infection (median absence 60 days, range 5–365 days).

**Table 4:** Characteristics for 79 WNV patients needing medical and paramedical services and other expenses by clinical syndrome <2 y after the acute phase of infection Quebec, Canada, 2012–2013*

Characteristic	WNF, n = 24	WNM, n = 17	WNE, n = 38†
Inpatient rehabilitation			
No. (%)	0	1 (6)	6(13)
No. days, median (range)	0	21	75 (30–120)
Physiotherapy			
No. (%)	2 (8)	3 (18)	6 (16)
No. visits, median (range)	20‡	28 (8–48)‡	32 (24–48)
Occupational therapy			
No. (%)	0	0	4 (11)
No. visits, median (range)	0	0	24 (3–32)
Speech therapy			
No. (%)	0	0	3 (8)
No. visits, median (range)	0	0	3 (1–3)
Home care			
No. (%)	1 (4)	0	2 (5)
No. hours, median (range)	1	0	2 (1–3)
Primary care physician visits			
No. (%)	15 (63)	12 (71)	25 (66)
No. visits, median (range)	2 (1–12)	4 (2–18)	2 (1–12)
Neurologist visits			
No. (%)	1 (4)	6 (35)	14 (37)
No. visits, median (range)	1	3 (2–4)	1 (1–4)
Medications			
No. (%)	7 (29)	10 (59)	15 (39)
Medical equipment			
No. (%)	1 (4)	1 (6)	6 (16)
No. using equipment, median (range)	3	2	2 (1–3)
Household chores			
No. (%)	0	0	2 (5)
Absence from work among workers			
No. (%)	6/8 (75)	15/15 (100)	16/16 (100)
No. days, median (range)	21 (5–60)	60 (7–180)	90 (30–365)§
Absence from work to care for a patient			
No. (%)	1 (4)	8 (47)	10 (26)
No. days, median (range)	3	5 (2–14)	7 (1–60)
Other personal expenses			
No. (%)	5 (21)	11 (65)	26 (68)

*WNE, West Nile encephalitis; WNF, West Nile fever; WNM, West Nile meningitis; WNV, West Nile virus. †Includes WNE (n = 19), meningoencephalitis (n = 18), and acute flaccid paralysis (n = 1). ‡Visits number missing for 1 participant. §Excluding 1 participant who reported stopping work because of his WNV infection.

Although 41% of patients reported outpatient medication expenses, they could not accurately recall the names and amounts of medication taken. Therefore, we did not compute medication costs.

### Direct and Indirect Costs

We determined direct and indirect costs according to illness severity (Table 5). WNE patients accounted for the largest proportion of total cost (median cost $21,332, IQR $12,131–$28,101) per participant compared with $8,124 (IQR $4,025–$13,631) for WNM patients (p = 0.0004) and $192 (IQR $20–$5,359) for WNF patients (p<0.0001). For WNE patients, costs were attributable mostly to hospitalization, which accounted for 65% of the total cost, followed by inpatient rehabilitation (20%). For WNM patients, indirect costs (47%), followed by hospitalization (38%), contributed to the largest proportion of total costs. For WNF patients, hospitalization (44%) and indirect costs (44%) contributed to the same proportion of total cost. Median indirect costs were significantly higher for WNM patients than for WNE (p = 0.004) or WNF (p = 0.0005) patients. Physician visit costs were also significantly higher for WNM patients than for WNE (p = 0.041) or WNF (p = 0.038) patients.

**Table 5:** Economic costs for 90 patients with WNV diseases by clinical syndrome, Quebec, Canada, 2012–2013*

Cost	WNF, n = 24	WNM, n = 18	WNE, n = 48†
Direct			
Mean (SD)	1,113 (2,048)	5,794 (7,625)	26,085 (28,363)
Median (IQR)	151 (20–685)	2,699 (2,483–7 703)	15,963 (7,881–28,065)
Total	27,271	106,167	1,257,249
Hospital			
Mean (SD)	892 (1,930)	4,221 (3,682)	19,259 (17,488)
Median (IQR)	0 (0–0)	2,333 (2,295–5,604)	15,839 (7,603–27,931)
Total	21,401	75,977	924,447
Emergency department visits			
Mean (SD)	55 (110)	110 (110)	110 (110)
Median (IQR)	55 (0–110)	110 (110–110)	110 (110–110)
Total	1,319	1,978	5,275
Inpatient rehabilitation			
Mean (SD)	0	1,019 (4,323)	6,004 (18,120)
Median (IQR)	0	0 (0–0)	0 (0–0)
Total	0	18,340	288,209
Outpatient readaptation‡			
Mean (SD)	74 (323)	289 (1,052)	480 (1,320)
Median (IQR)	0 (0–0)	0 (0–0)	0 (0–0)
Total	1,780	5,194	23,068
Physician visits§			
Mean (SD)	74 (106)	180 (185)	89 (124)
Median (IQR)	41 (0–93)	126 (0–277)	60 (0–136)
Total	1,781	3,242	4,279
Additional costs¶			
Mean (SD)	41 (104)	80 (66)	250 (673)
Median (IQR)	0 (0–0)	100 (0–100)	100 (0–100)
Total	990	1,435	11,971
Indirect costs (absence from work)#			
Mean (SD)	896 (2,198)	5,169 (6,116)	3,454 (9,101)
Median (IQR)	0 (0–556)	2,550 (650–9,000)	0 (0–3,374)
Total	21,506	93,042	165,772
Total costs			
Mean (SD)	2,009 (2,904)	10,963 (9,072)	29,539 (31,067)
Median (IQR)	192 (20–5,359)	8,124 (4,025–13,631)	21,332 (12,131–28,101)
Total	48,777	199,209	1,423,021

*Costs are in Canadian dollars. Total costs are the sum of all category costs. Mean and median costs are expressed as per-patient cost for all participants (including patients with 0 costs). Total is the costs sum for all participants. IQR, interquartile range; WNE, West Nile encephalitis; WNF, West Nile fever; WNM, West Nile meningitis; WNV, West Nile virus. †Includes WNE encephalitis (n = 28), meningoencephalitis (n = 18), and acute flaccid paralysis (n = 2). ‡Includes physiotherapy, occupational therapy, speech therapy, and home care costs. §Includes primary care physician and neurologist consultations costs. ¶Includes medical equipment purchase, recruitment for household chores costs, and other personal expenses. #Includes participants’ and families’ missed work costs.

During 2012, a total of 124 symptomatic WNV patients were reported in the province of Quebec, and during 2013, a total of 31 symptomatic WNV patients were reported. Based on our study, the estimated total cost of these cases was ≈$1.7 million for 2012 and ≈$430,000 for 2012. We determined the estimated total cost by category (Table 6). For both years, WNF accounted for <5% of the total, and WNM accounted <10% of the total and WNE accounted for >85% of extrapolated costs.

**Table 6:** Total extrapolated costs for WNV cases by clinical syndrome, Quebec, Canada, 2012–2013*

Cost	2012, n = 124									2013, n = 31							
	WNF			WNM			WNE			WNF			WNM			WNE	
	No.	Cost		No.	Cost		No.	Cost		No.	Cost		No.	Cost		No.	Cost
Hospitalization	11	61,644		23	54,556		62	982,018		1	5,604		7	16,604		16	253,424
ED visit	19	2,090		24	2,640		62	6,820		4	385		7	770		17	1,870
Inpatient rehabilitation	0	0		1	24,453		8	304,583		0	0		0	0		2	83,515
Outpatient readaptation†	3	2,818		4	2,528		9	34,286		1	519		1	737		2	9,401
Physician visit‡	24	1,900		17	4,004		37	4,158		4	350		5	1,168		10	1,140
Additional§	8	792		16	1,600		45	4,521		1	146		5	467		12	1,240
Indirect¶	11	14,054		21	68,267		23	104,625		2	2,589		6	19,911		6	28,688
Total	38	83,298		24	158,048		62	1,441,010		7	9,593		7	39,657		17	379,277

*Costs are in Canadian dollars. Total costs are the sum of all category costs. ED, emergency department; WNE, West Nile encephalitis; WNF, West Nile fever; WNM, West Nile meningitis; WNV, West Nile virus. †Includes physiotherapy, occupational therapy, speech therapy, and home care costs. ‡Includes primary care physician and neurologist consultations costs. §Includes medical equipment purchase, recruitment for household chores costs, and other personal expenses. ¶Includes participants’ and families’ missed work costs.

## Discussion

In this study, we estimated direct and indirect costs of WNV disease cases in Quebec, Canada, up to 2 years after the acute phase of WNV disease. We found that costs varied considerably according to disease manifestations. Patients with WNE accounted for the largest proportion of the total cost, which could be attributable mainly to their worse hospital course, including severe clinical manifestations, longer stay in the hospital, admission to the intensive care unit, and complications (*6*). When we compared WNM with WNE patients, we found that WNE patients had lower indirect costs (absence from work), which could be explained by their older age (≈70% were retired at the time of their WNV infection vs. only 17% of patients with WNM).

Although most WNM patients were hospitalized, hospital costs accounted for only 38% of the total cost. WNM is generally associated with a favorable outcome and shorter hospital stay. However, in our study, WNM patients required more physician visits after hospitalization than patients with WNE or WNF. WNF patients accounted for only 3% of the total cost, which was mostly associated with the 5 hospitalized case-patients. For these patients, the median cost of hospitalization was similar to that for the 17 hospitalized WNM patients (p = 0.901) (Table 7). In our previous study (*6*), we showed that hospitalized WNF patients had similar demographic and clinical profiles as WNE patients, and we assumed that some of them could have undetected mild neuroinvasive disease because 3 of 5 hospitalized WNF patients did not have a lumbar puncture. However, half of WNF patients had consulted only a clinic, and costs associated with their initial care were not available. Thus, the total WNF costs could be underestimated.

**Table 7:** Median economic costs of WNV diseases per patient by clinical syndrome, Quebec, Canada, 2012–2013*

Cost	Disease, no. patients; cost (IQR)		
	WNF	WNM	WNE†
Hospitalization	5; 5,604 (2,295–5,604)	17; 2,372 (2,295–5,604)	47; 15,839 (7,603–27,931)
ED visit	12; 110 (110–110)	18; 110 (110–110)	48; 110 (110–110)
Inpatient rehabilitation	0, 0	1; 18,340 (18,340–18,340)	6; 39,301 (26,200–78,602)
Outpatient readaptation‡	2; 890 (200–1,580)	3; 632 (100–4,462)	7; 3,792 (2,540–4,661)
Physician visit§	15; 80 (42–160)	13; 231 (126–280)	29; 111 (71–152)
Additional¶	5; 100 (100–340)	12; 100 (100–125)	35; 100 (100–115)
Indirect (absence from work)#	7; 1,268 (961–5,434)	16; 3,200 (1,259–9,418)	18; 4,500 (3,000–8,653)

*Costs are in Canadian dollars. Median costs are expressed as per-patient cost on the basis of participants who incurred costs in each category (excluding patients with 0 costs). ED, emergency department; IQR, interquartile range; WNE, West Nile encephalitis; WNF, West Nile fever; WNM, West Nile meningitis; WNV, West Nile virus. †Includes WNE (n = 28), meningoencephalitis (n = 18), and acute flaccid paralysis (n = 2). ‡Includes physiotherapy, occupational therapy, speech therapy, and home care costs. §Includes primary care physician and neurologist consultations costs. ¶Includes medical equipment purchase, recruitment for household chores costs, and other personal expenses. #Includes participants, and families, missed work costs.

These results are similar to those reported by Staples et al. (*8*), who estimated the initial and 5 years post-WNV infection costs among hospitalized patients in Colorado, USA, during 2003. These authors reported that initial costs were higher for AFP and WNE patients, and long-term costs were higher for AFP and WNM patients. However, this study focused on hospitalized case-patients and might not be a true reflection of all reported WNV patients. Two other studies have evaluated the economic burden of WNV outbreaks in the United States, but both evaluated only initial costs (*7*,*9*).

When we extrapolated our results to all reported WNV cases in the province of Quebec, we estimated that WNV infections could cost ≈$1.7 million during an epidemic, such as during 2012; during a low activity year, such as 2013, the cost could be ≈$430,000. These results are consistent with those of the cost-effectiveness simulation study of Bonneau et al. (*10*), which estimated total direct and indirect cost of ≈$400,000 during a hypothetical year of low activity (25 cases of WNV infection) and >$13 million during a hypothetical year of high activity (840 cases of WNV infection). In that analysis, direct costs included hospitalization, rehabilitation, and outpatient consultations, and indirect costs included productivity losses caused by absence from work and death.

Our study had several limitations. Although participation rate was high and participants were similar to nonparticipants, analyses by clinical categories were based on a small number of cases. However, this number is similar to those for 3 studies in the United States (*7*–*9*). Some costs, such as initial consultations in private practice and medication expenses during follow-up, were not included because they were unavailable or they lacked precision. However, these costs accounted for a small proportion of total cost. Recall biases were also possible for costs incurred during follow-up.

Calculation of productivity losses varies between studies. Because of ethical issues related to the evaluation of productivity losses for a dead person (e.g., is an old person less worthy than a young person because of the fact that he or she is retired or less productive?) (*15*), we decided not to include the indirect costs associated with death in our results. This decision resulted in an underestimation of the WNV economic burden, particularly for WNE patients, because 10 of them plus 1 WNM patient died during their initial hospitalization. Grosse et al. (*17*) estimated for the United States the productivity value by age and sex on a daily, annual, and lifetime basis (in 2007 US dollars). Such productivity tables are not available for Canada and Quebec. However, when we used daily production values and age and sex distribution of Grosse et al. (*17*) for our cases, we estimated the loss of productivity caused by WNV deaths over a 2-year period after the infection. To take into account time preference, we applied a discount rate of 3%, 5%, and 8% and converted the results to 2013 Canadian dollars on the basis of methods suggested by Montmarquette and Scott (*18*) and Tchouaket et al. (*19*). These estimates ranged from $467,000 (3% discount rate) to $589,000 (8% discount rate) and would represent a 35% increase over costs we calculated (Table 5).

In comparison, Zohrabian et al. (*7*) calculated lifetime lost productivity for persons who died, and this productivity loss represented half of the total costs of illness. Staples et al. (*8*) valued productivity losses for those who died but not for older persons who were retired at the time of their illness, and evaluation led to lower indirect costs for WNE than for WNM. In their simulation study, Bonneau et al. (*10*) showed that nearly 70% of total costs were attributable to indirect costs (deaths and absence from work).

In summary, we found that the overall cost of WNV infection in Quebec was ≈1.7 million for 2012 (24 symptomatic cases) and ≈430,000 for 2013 (31 symptomatic cases) and that costs were significantly higher for patients who had more severe forms of disease. Our study provides information to government and public health organizations to make informed decisions regarding preventive and intervention programs for WNV infection. Public health monitoring of costs, both direct and indirect, associated with different clinical manifestations of infectious diseases is essential to enable adequate planning for public health policies and infectious diseases prevention and control programs.
